# Evaluation of Infrared Thermography for the Detection of Footrot and White Line Disease Lesions in Dairy Sheep

**DOI:** 10.3390/vetsci8100219

**Published:** 2021-10-05

**Authors:** Athanasios I. Gelasakis, Aphrodite I. Kalogianni, Marios Moschovas, Eirini Tsimpouri, Theodoros Pnevmatikos, Ioannis Bossis, Georgios Arsenos, Panagiotis Simitzis

**Affiliations:** 1 Laboratory of Anatomy and Physiology of Farm Animals, Department of Animal Science, School of Animal Biosciences, Agricultural University of Athens (AUA), Iera Odos 75 str., 11855 Athens, Greece; afrokalo@aua.gr (A.I.K.); moschovas@aua.gr (M.M.); stud215093@aua.gr (E.T.); stud215125@aua.gr (T.P.); 2 Laboratory of Animal Husbandry, Department of Agricultural Sciences, School of Agriculture, Forestry and Natural Resources, University Campus, Aristotle University of Thessaloniki (AUTh), 54124 Thessaloniki, Greece; bossisi@agro.auth.gr; 3 Laboratory of Animal Husbandry, School of Veterinary Medicine, Faculty of Health Sciences, University Campus, Aristotle University of Thessaloniki (AUTh), 54124 Thessaloniki, Greece; arsenosg@vet.auth.gr; 4 Laboratory of Animal Breeding and Husbandry, Department of Animal Science, School of Animal Biosciences, Agricultural University of Athens (AUA), Iera Odos 75 str., 11855 Athens, Greece; pansimitzis@aua.gr

**Keywords:** infrared thermography, point-of-care diagnostics, dairy sheep, foot-related lameness, footrot, white line disease, binary regression analysis, ROC curves, sensitivity, specificity

## Abstract

The objectives of this study were to investigate temperature distribution at the sheep hoof and evaluate the reliability and diagnostic performance of infrared thermography (IRT) for the detection of footrot and white line disease (WLD) lesions in intensively reared dairy sheep. Hoof lesions were clinically assessed, and IRT was used to measure temperature distribution on hoof superficial tissue in 600 multiparous ewes. Binary regression models were developed and validated, and receiver operating characteristic curves were estimated to assess the predictive value and diagnostic performance of IRT for the detection of hoof lesions. The most sensitive prediction model for the detection of IFR was based on the difference between ambient and hoof heel temperature (sensitivity: 83.3%, specificity: 47.8%, and threshold value: 6.5 °C), whereas the most specific prediction model was based on the difference between ambient and coronary band temperature (sensitivity: 51.9%, specificity: 79.7%, and threshold value: 11.3 °C). In the case of WLD, the diagnostic performance of IRT had limited predictive value. IRT could be a useful tool for hoof health screening in dairy sheep. However, it must be cautiously adapted in cases where environmental, operating, and operator variables are not effectively controlled.

## 1. Introduction

In dairy sheep, foot-related lameness is a significant welfare-challenging issue. Particularly in intensive farming systems, an early, quick, and accurate diagnosis of lameness-related foot lesions has emerged as a field of interest for veterinarians, consultants, and farmers. In these farming systems, footrot and white line disease (WLD) have been recognized among the most significant infectious and noninfectious foot-related lameness causes, respectively [1,2,3,4], with their prevalence in Greece being ca. 8% (from 2 to 14%) and 38% (from 30 to 51%), respectively [5]. Footrot is the most detrimental lameness-related bacterial foot disease with global spread [6]. It is exceptionally contagious due to its extensive horizontal transmission among animals [7,8], and it is primarily caused by *Dichelobacter nodosus*, whereas the opportunistic presence of *Fusobacterium necrophorum* deteriorates the severity of lesions [9]. Footrot is associated with severe inflammation and a gradation of underrunning of the hoof, leading to an extremely painful situation with the complete separation of the horn from the hoof matrix in advanced cases [10]. WLD refers to the varying degrees detachment of the hoof wall from the laminar corium. Although the etiology of WLD has not yet been elucidated and is not considered a cause of lameness per se, it predisposes them to foot infections due to debris accumulation and, in advanced cases, to abscess formation (white line abscess), pain, and lameness [7,11]. Foot lesions causing foot-related lameness in sheep have been associated with adverse effects (i) on animal welfare, causing pain and distress; (ii) on animal productivity by reducing the weight gain, wool quality, and milk yield; and (iii) on farm profitability due to production losses and increased treatment costs [3,7,12,13]. Currently, clinical examination and exploratory foot-trimming are performed for the diagnosis of footrot and WLD; however, they require restraint of the animals and experienced foot-trimmers, while the criteria to select animals for exploratory foot-trimming (other than lameness) are lacking [11,14]. Subsequently, for the accurate assessment of foot health status, all animals need to be foot-trimmed on a regular basis [1]. Although routine foot-trimming twice per year is rather a necessary prophylactic measure in permanently housed sheep, where hooves are not naturally worn down, it does not ensure the timely diagnosis of underlying hoof lesions, whereas, in some cases, it could be associated with an increased risk of footrot transmission and lameness, as observed in meat breeds of sheep [15]. Consequently, the use of thermal imaging of the hoof and the development of prediction models to detect underlying lesions could offer a rapid, on-site, noninvasive diagnostic tool and a sustainable component for precise foot-health management (e.g., targeted foot-trimming, evidence-based preventive measures, and reduction in the use of antibiotics and footbath solutions).

The notion of developing and validating technologies with the potential to serve as point-of-care diagnostics is not new; however, the last two decades has urged the idea of utilizing infrared thermography (IRT) in animal practice [16,17]. IRT is a remote-sensing technology that measures the amount of infrared energy emitted, reflected, or transmitted by objects and converts it into images. These images display the temperature distribution of the captured surfaces [18,19] via the detection of infrared radiation from the objects’ surfaces, which range from 750 nm to 1 mm, and is invisible to the human eye [16]. It is based on the principle that objects with temperatures above absolute zero (−273.15 °C) generate radiant heat in the infrared spectrum and emit radiation, creating an electromagnetic spectrum that may be absorbed by other objects around them [20]. The advantages of IRT applications over typical thermometers are (i) its noninvasive application without a demand for stressful restraining and handling of animals, (ii) the rapid measurement of the temperature and changes thereof in any part of the animal’s body, and (iii) the temperature benchmarking capacity over wide body surfaces via imaging of the temperature distribution [21]; moreover, IRT does not affect the temperature of the object surface by conduction or convection, in contrast to thermal sensors [22].

Most of the applications of IRT in animal production refer to the allocation of sites on the body surface with increased temperatures [23] to detect (i) fever [24]; (ii) stress [25,26,27]; (iii) inflammation sites due to infectious and noninfectious diseases and injuries [28,29,30,31,32]; and (iv) estrous in cattle, sheep, and pigs [33,34,35].

Recently, IRT has been evaluated as a prediction tool for the diagnosis of foot lesions, mainly in dairy cows [36,37,38,39], with promising results, whereas, in sheep, the thermal imaging of hooves has been used in the past but exclusively in meat breeds for the detection of lameness-related lesions [40]. Increased feeding and metabolic rates and the permanent housing of intensively reared dairy ewes are factors with the potential to affect hoof health and temperature, modifying the efficiency of thermal imaging when compared to meat sheep. In any case, extrapolation of the results from studies on dairy cows or meat sheep is not feasible, and the possibility of developing universally applied prediction models, with adequate sensitivity and specificity across farm animal species and production systems, is limited. Therefore, studies on the potential applications of IRT need to be extended and separately validated in various farm animal species and reared under different management schemes for different foot pathologies.

Therefore, the objective of the present study was to describe the temperature distribution at the coronary band, the heel, and the sole of the sheep hoof and evaluate the reliability and diagnostic performance of IRT for the detection of footrot and WLD lesions in intensively reared dairy sheep.

## 2. Materials and Methods

### 2.1. Farm and Animal Selection

Thirty intensive sheep farms with approximately 10,630 ewes in total were included in the study. The farm characteristics were described in detail by Moschovas et al. [5]. In brief, they were medium-sized (between 250 and 400 ewes), high-producing (>320 kg milk/ewe/210 days of lactation) farms located in plain areas with considerable investments on infrastructures and equipment to support zero-grazing, intensive management. Animals were permanently housed in deep litter and continuously supplemented indoors with concentrates, lucerne hay, minerals, and vitamins to meet their nutritional demands. Foot care protocols included foot-trimming once per year, whereas foot bathing was not applied in any case. A designated questionnaire was developed to gather data regarding the farm structure; livestock traits and management; labor; infrastructures; feeding and nutrition; reproduction; biosecurity and hygiene measures; disease control protocols; preventive veterinary medicine; and the flock health status, with emphasis on the foot health status (etiology, epizootiology, and control measures of foot-related lameness and associated disorders).

A multivariable statistical approach (principal component analysis and cluster analysis) was used to define the typology of the farming systems as the basis for the subsequent selection of representative farms, as detailed by Moschovas et al. [5]. The farms were classified into two clusters, and three farms per cluster were randomly selected. From each of the six farms, 100 multiparous ewes were selected if 20–50 days post-lambing and belonging to one of the following three breeds: Chios, Lacaune, and Frizarta. The ewes (*n* = 600) were enrolled in a cross-sectional study from October to December 2020 to record and assess the occurrence, topography, and severity of foot lesions.

### 2.2. Thermal Imaging and Data Recording

**Figure 1:**
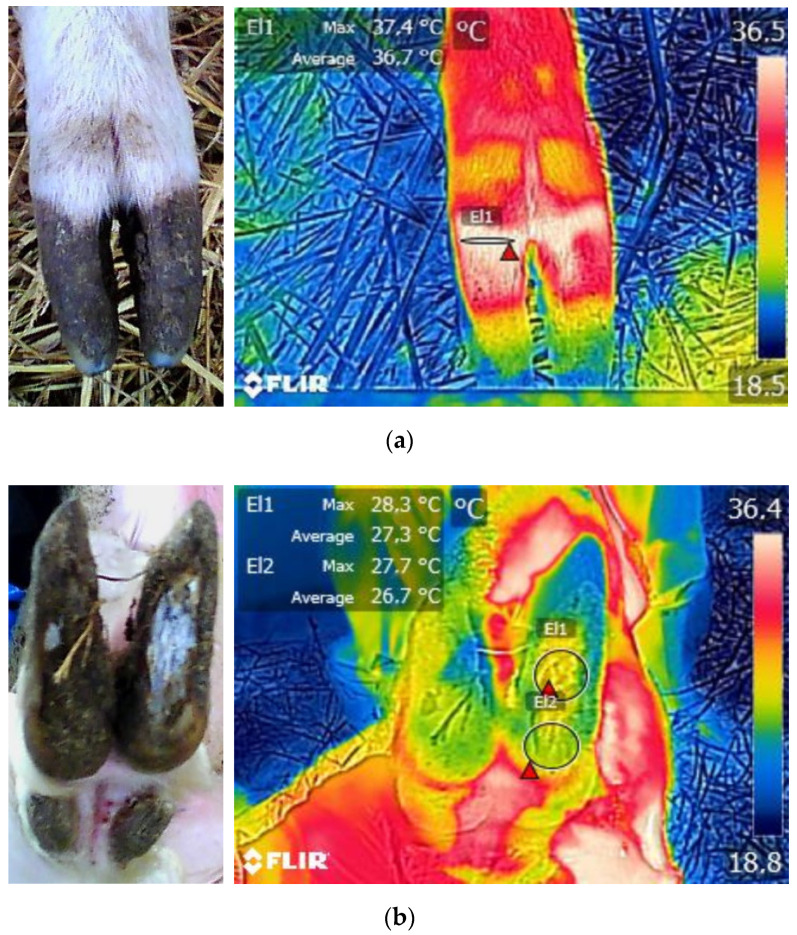
Temperature measurements at: (**a**) the coronary band (T1 = El1), (**b**) the hoof sole (T2 = El1), and the hoof heel (T3 = El2).

Thermographic images of the hooves from individual ewes were captured from the dorsal and plantar views almost vertically from a 50-cm approximate distance using an infrared camera (FLIR E8-XT, FLIR Systems Inc., Wilsonville, OR, USA). To avoid operator-derived inconsistencies, all the images were captured by the same trained operator using a predefined protocol as regards the settings and the procedure (e.g., distance and angle of capture). The thermal sensitivity, noise equivalent temperature difference (NETD), accuracy, emissivity, and resolution of the camera were 0.06 °C, <60 mK, ±2 °C, 0.95, and 320 × 240, respectively. Moreover, to avoid the disrupting effects of direct exposure to the forementioned factors, thermal images were captured inside the barn without exposure to direct sunlight, wind, and increased humidity and always after the gentle removing of debris from hooves. Routine foot-trimming was performed by experienced foot-trimmers and was supervised by a veterinarian who also performed the physical examination, assessed the body condition score (BCS, 1–5: 1 = emaciated; 5 = obese with 0.25 increments), and recorded the foot-health status. Footrot and WLD occurrence were defined by a clinical appraisal of the lesions revealed during trimming. Underrunning of the hoof and necrosis of the underlying tissues, followed by the separation and deformation of the horn, were used to define a footrot case in our study. White line disease was defined as the occurrence of defects at the junction between the abaxial hoof wall and the sole, followed by a various extend separation of the inner layer of the hoof wall from the laminae. All images were processed with Flir Tools software (v 5.X) to record the ambient temperature (AT) and maximum temperatures in three predefined hoof sites: (i) the coronary band (T1), (ii) the hoof heel (T2), and (iii) the hoof sole (T3) (Figure 1). For measuring the maximum temperatures, the circular and ellipse selection tools were chosen to outline the limits of the relative hoof sites.

Differences between the maximum temperatures and AT were calculated by subtraction (DT-1, DT-2, and DT-3 for the coronary band, hoof heel, and hoof sole, respectively). Ear tags and lactation numbers of the animals were also available. An Excel database with a total of 4800 records at the hoof level was developed, and an equal number of thermographic images were analyzed.

### 2.3. Statistical Analyses

For the statistical analyses, SPSS v23 software (IBM Corp., Armonk, NY, USA) was used, and the statistical significance was set at the 0.05 level. The internal consistency (reliability) of IRT was estimated by calculating the Cronbach’s alpha coefficient, and the dimensionality was estimated using a principal component analysis [41].

Descriptive statistics were calculated (mean ± standard deviation for continuous variables and frequencies for categorical variables). Data were also analyzed to assess the contribution of the lactation number, BCS, and hoof temperature in predicting the occurrence of footrot and WLD of the *i*th ewe in the *j*th farm. Two sets (referring to the two outcome variables—namely, footrot and WLD occurrence) of three binary logistic regression models (referring to the three hoof sites where the temperature differences were estimated) were used as described below:

Logit [Pr (Y_ij_ = 1)] = β_0_ + β_1_*LAC_ij_ + β_2_*BCS_ij_ + β_3_ − β_5_*DTEMP_ij_ + ε_ij_

where Y = outcome variable (occurrence of footrot and WLD at the hoof level); β_0_ = constant; β_1_ = coefficient of lactation number (LAC) (4 levels: 2nd, 3rd, 4th, and ≥5th lactation); β_2_ = coefficient of the body condition score (BCS); β_3_ – β_5_ = coefficients of DT-1, DT-2, and DT-3 (DTEMP); and ε = random residual error. The backwards stepwise method was used for the selection of the predictor variables in the models, with the *p*-value being set at the 0.1 level for this purpose.

The Hosmer–Lemeshow (H-L) test, Omnibus test of coefficient, and Nagelkerke R^2^ indices were used to assess the goodness-of-fit and the amount of variations explained by each individual model. Additionally, the internal validity of the models was evaluated by split-half cross-validation [42]. The regression coefficients, standard errors, 95% confidence intervals, and *p*-values for the models and predictors were calculated. Receiver operating characteristic (ROC) curves were drawn, and the areas underneath them (AUC, c-statistic) were calculated to compare the diagnostic performance of thermal imaging; the optimal efficiency thresholds, as well as the sensitivity (Se) and specificity (Sp) values, when the predicted probabilities were considered for IRT and WLD lesions, were estimated.

## 3. Results

### 3.1. Descriptive Statistics

**Figure 2:**
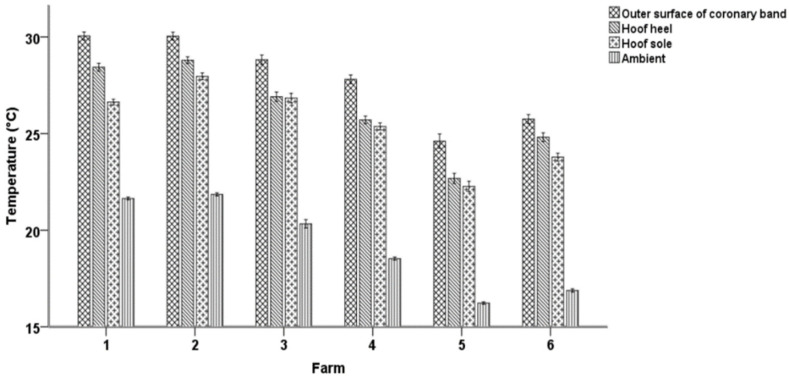
Mean temperature values and error bars for (i) the coronary band, (ii) hoof heel, and (iii) hoof sole and the ambient temperatures in the six studied farms.

Overall, the prevalence of footrot and WLD lesions at the hoof level was 1.2% (56/4800) and 7.9% (377/4800), respectively. The mean values of T1, T2, and T3 and of DT-1, DT-2, and DT-3 were 27.9 ± 4.14, 26.2 ± 3.73, and 25.5 ± 3.48 °C and 8.6 ± 3.34, 7.0 ± 2.81, and 6.2 ± 2.60 °C for the coronary band, the hoof heel, and the hoof sole, respectively. Figure 2 presents the mean values of the respective temperatures and ATs at the six farms included in the study.

**Figure 3:**
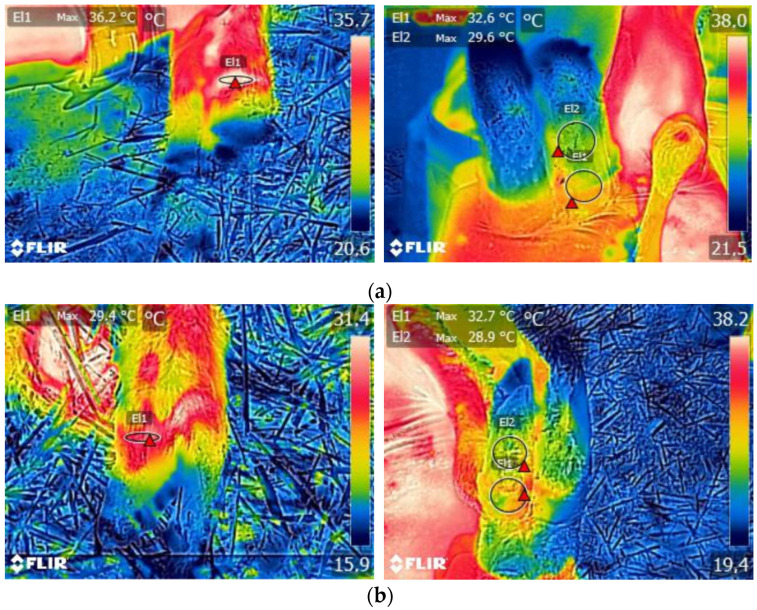
Maximum temperature measurements at the coronary band (T1 = El1), the hoof sole (T2 = El1), and the hoof heel (T3 = El2) in animals with (**a**) footrot and (**b**) white line disease lesions.

In ewes with footrot, the mean values of the maximum hoof temperatures were 30.0 ± 4.48, 28.2 ± 4.15, and 27.2 ± 3.81 °C at the coronary band, the hoof heel, and the hoof sole, respectively (Figure 3a), whereas, for the animals without footrot, they were 27.8 ± 4.13, 26.2 ± 3.72, and 25.5 ± 3.47 °C, respectively. In the case of WLD, the respective values were 28.5 ± 4.05, 26.1 ± 3.57, and 25.7 ± 3.32 °C for ewes with WLD lesions (Figure 3b) and 27.8 ± 4.14, 26.2 ± 3.72, and 25.5 ± 3.47 °C for ewes without.

### 3.2. Consistency of IRT

**Figure 4:**
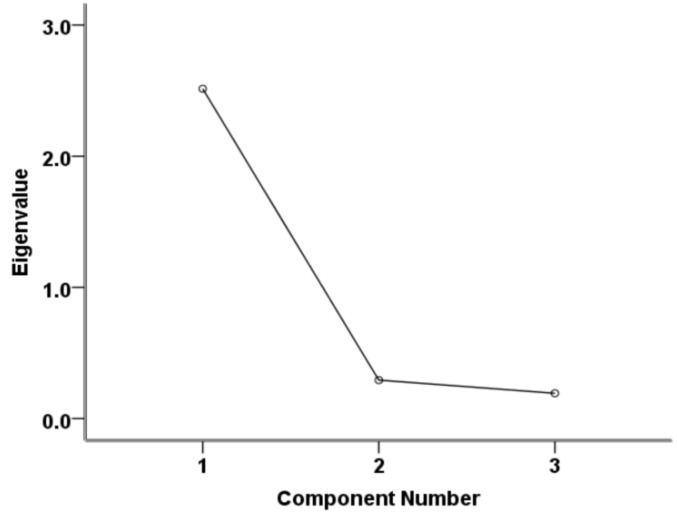
Scree plot of the components’ eigenvalues as estimated by the principal component analysis.

The Cronbach’s alpha coefficient was 0.932, indicating a sufficient reliability of IRT for the purposes of the study. Furthermore, only one principal component was found by PCA to have an eigenvalue > 1 (ca. 2.5), accounting for 83.8% of the total variance and, therefore, satisfying the assumption of one-dimensionality of the temperature measurements (Figure 4).

### 3.3. Goodness-of-Fit and Performance of the Models for the Diagnosis of Footrot and WLD

In all the models, the Hosmer and Lemeshow tests were statistically insignificant, while the Omnibus tests of coefficients were significant, indicating that the models provided a good fit to the data and were predictive, respectively (Table 1), whereas the Nagelkerke R^2^ varied from 0.032 to 0.043 and from 0.012 to 0.013 for the models predicting footrot and WLD lesions, respectively.

In all the footrot prediction models, the differences between the measured hoof sites and ATs were statistically significant predictors (*p* < 0.001 for DT-1; *p* < 0.01 for DT-2 and DT-3), while the lactation number and BCS were not (Table 2). A one-degree Celsius increase in DT-1, DT-2, and DT-3 was associated with an increased likelihood of footrot occurrence by 1.23, 1.21, and 1.25 times, respectively.

Similarly, in the case of WLD prediction models, the predictive values of DT-1 and DT-3 were significant (*p* ≤ 0.01) (Table 2); a one-degree Celsius increase in DT-1 and DT-3 was associated with increased likelihood of WLD occurrence by 1.08 and 1.10 times, respectively. Moreover, BCS had a significant effect in the model, using DT-1 as a predictor for WLD lesions (*p* < 0.05); a one-degree increase in BCS was associated with a decreased likelihood of WLD occurrence by 1.75 times.

All the models were successfully validated using split-half cross-validation; one exception was the model that used DT-2 as a predictor of WLD lesion occurrence (Table 3).

**Figure 5:**
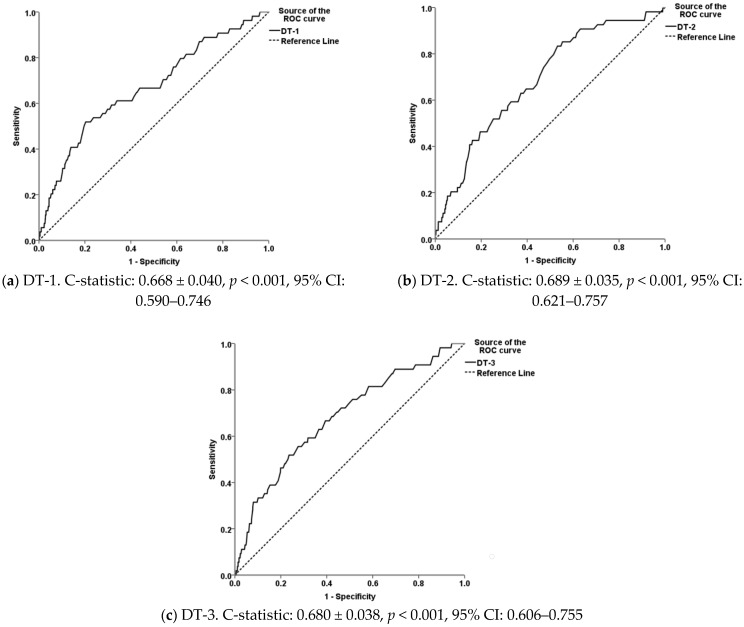
Receiver operating characteristic (ROC) curves illustrating predicted probabilities and the diagnostic performances of the differences between the ambient temperature and the maximum temperatures at (**a**) the coronary band (DT-1), (**b**) the hoof heel (DT-2), and (**c**) the hoof sole (DT-3) as regards footrot; mean values of the areas under the ROC curves (C-statistic ± standard error), statistical significance, and 95% confidence intervals (CI) are also presented.

**Figure 6:**
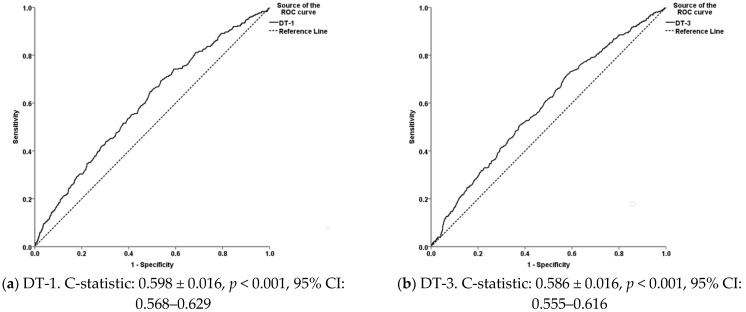
Receiver operating characteristics (ROC) curves illustrating the predicted probabilities and the diagnostic performances of the differences between the ambient temperature and the maximum temperatures at (**a**) the coronary band (DT-1) and (**b**) the hoof sole (DT-3) as regards white line disease. Mean values of the areas under the ROC curves (C-statistic ± standard error), statistical significance, and 95% confidence intervals (CI) are also presented.

Moreover, the c-statistic values varied from 0.668 to 0.689 for the footrot prediction models and from 0.586 to 0.598 for the respective WLD models (Figure 5 and Figure 6) and were statistically significant in all cases.

Table 3 summarizes the optimal efficiency thresholds for the predicted probabilities and DTs, sensitivity, and specificity, as estimated by the binary regression models, considering: (i) the total dataset, (ii) the training sample, and (iii) the validation sample. Among the footrot prediction models, the highest sensitivity (83.3%; 76.0% and 89.7% for the training and validation samples, respectively) was observed when DT-2 was considered as the predictor (DT-2 threshold value: 6.5 °C). The highest specificity (79.7%; 80.1% and 81.0% for the training and the validation samples, respectively) was found when DT-1 was used as the predictor (DT-1 threshold value: 11.3 °C). For the WLD prediction models, the highest sensitivity (73.1%; 75.9% and 62.3% for the training and the validation samples, respectively) was estimated by the model using DT-3 as the predictor (DT-3 threshold value: 6.4 °C) and the highest specificity (51.0%; 49.4% and 44.7% for the training and the validation samples, respectively) by the model using DT-1 as the predictor (DT-1 threshold value: 3.6 °C) (Table 3).

## 4. Discussion

The results showed that IRT is a reliable tool for measuring the temperatures at the coronary band, the hoof heel, and the hoof sole in sheep. Considering the available literature, this is the first study of temperature values at these hoof sites in dairy sheep with thermal imaging that was assessed for its consistency. Another innovative aspect of this study is the use of differences between temperatures at the selected hoof sites and the AT to predict the occurrence of footrot and WLD lesions. The latter confirmed the notion of a high diagnostic value of IRT for detecting hoof lesions. All the models were fairly predictive and were efficiently validated (except for the model predicting WLD lesions using DT-2 as the predictor). However, their performances varied according to the predicted lesions (footrot or WLD) and among the studied hoof sites.

Factors that significantly affect the reliability and performance of IRT and, therefore, need to be avoided during thermal imaging of the hoof are: (i) direct exposure to sunlight, (ii) high humidity level, and (iii) convective heat loss (e.g., wind and dirt on the surface), as the radiation measures and thermal imaging thereof are not only a function of the object’s temperature but, also, of its emissivity and conductivity [20]. All these factors were considered in the study design. Additionally, to overcome the potential confounding effect of AT, we selected to use the differences between the AT and the maximum hoof temperatures at the selected hoof sites as predictors; this was considered necessary, as, in all the studied farms, the maximum temperatures at the selected hoof sites followed the same pattern (increasing or decreasing) with the AT, implying that AT was associated with the absolute values of the hoof temperatures. By addressing these factors, it was possible to maintain a sufficient reliability and performance, as indicated by the calculated Cronbach’s alpha coefficient and the models’ performance metrics, respectively.

In the present study, a large database of captured images was developed, and their detailed assessment revealed that the temperature at the coronary band was the highest among the three hoof sites. This was an expected finding, as it is well-known that the coronary band overlies tissues that are rich in vessels to support the increased blood flow demands for the distribution of nutrients in the hoof. Additionally, from the poorer perspiration, the hoof heel and the hoof sole are expected to have lower temperatures due to the outer keratinized horn layer covering themselves; nevertheless, in hooves with footrot lesions, the temperature was increased by almost 2 °C, even in the hoof heel and the hoof sole. On the contrary, much lower temperature differences were observed in hooves with WLD lesions, possibly due to the absence of a noticeable inflammatory response.

In general, the suitability of different hoof sites needs to be assessed prior to their consideration in thermal imaging studies in sheep. Currently, relevant studies are scarce, and the mapping of temperature distribution at the hoof is not available. In the real world, uncontrolled environmental conditions, as well as physiological, pathological, and, possibly, genetic factors, influence the hoof temperature and make the suggestion of universally accepted hoof sites for the diagnosis of hoof lesions a complicated task. Another aspect that needs to be considered is the fact that it is not possible to exploit the noninvasive potential of thermal imaging for all the hoof sites. For example, thermal imaging without lifting the feet is possible for the coronary band but not for the hoof heel and the hoof sole.

The differences between the studied hoof sites and the AT varied from 6.2 to 8.6 °C. All the studied farms were at plain regions with a temperate climate, while the study was undertaken during autumn. For the use of the developed models of this season, and on a regular basis throughout the year, their across-seasons validation is important in order to determine whether the estimated differences between the studied sites and the AT remain constant; if they are modified, it is likely that the diagnostic performance and applicability of the models are not adequate, and season-specific models need to be developed.

Footrot is associated with severe inflammation [7,8,10], explaining the higher hoof temperatures in footrot-affected hooves and the significant predictive capacity of DT-1, DT-2, and DT-3 for the detection of footrot lesions occurrence. Additionally, the single model that was not validated was the one that used DT-2 to predict WLD lesion occurrence (Table 3). The hoof heel does not present a significant anatomical or functional relationship with white line, contrary to the hoof sole (white line is the anatomical joint between the horn of the hoof wall and that of the sole) and the coronary band (towards which the WLD lesions expand); thus, the absence of significant alterations of the hoof heel temperature due to the occurrence of WLD lesions was not an unexpected finding.

The optimal efficiency thresholds were estimated by estimating the ROC curve coordinates (predicted probabilities) for which the combination of the sensitivity and specificity values were maximized. When the threshold of DT-2 was set at 6.5 °C, the model provided the highest sensitivity for the diagnosis of footrot (ca. 83.0%) but a low specificity (ca. 48.0%); on the other hand, when the threshold of DT-1 was set at 11.3 °C, the specificity for the diagnosis of footrot was ca. 80.0% and the sensitivity ca. 52.0%. Therefore, it can be suggested that a combination of temperature measurements at both the coronary band and the hoof heel is the most appropriate approach for the sensitive and specific diagnoses of footrot. Nevertheless, a limitation in our study, which could have led to an underestimation of the diagnostic performance of thermal imaging for the detection of footrot in the studied hoof sites, is the low prevalence of active footrot lesions; future studies for external validation of the models in farms with a high prevalence of footrot would be of value to address this limitation.

For the detection of WLD lesions, the sensitivity of the model using DT-3 as the predictor was ca. 73.0%, whereas none of temperature differences between the studied hoof sites and AT produced satisfying results as regards the specificity of the models. Based on these findings, it could be assumed that the diagnostic performance of thermal imaging at the studied hoof sites is higher for the detection of footrot lesions than for WLD lesions. Severe and extended inflammation of the hoof underlying tissues in the case of footrot is consistent with this finding, whereas, in the case of WLD, the absence of inflammation at the measured sites is likely; this is a finding that adds to the scarce information regarding the pathogenesis of the disease. In any case, the thermal imaging of other hoof regions (e.g., abaxial hoof wall and white line before and after exploratory foot-trimming) could possibly reveal hoof sites with a better diagnostic performance for the detection of WLD lesions. Another finding adding to our knowledge regarding WLD is that the animals with higher BCS had a decreased likelihood of being diagnosed with WLD. This could imply that WLD is associated with the feeding efficiency and the overall nutritional status of animals. However, this is a speculation, and another study design is necessary to confirm this hypothesis and reveal the underlying mechanisms.

The mean values of the maximum hoof temperatures at the studied hoof sites are consistent with the maximum hoof temperature of healthy ewes as calculated by Byrne et al. [40] in three meat breeds (Texel, Suffolk, and Belclare and their crosses). However, apart from the different productive orientations and the lower number of the animals included in the latter study (*n* = 103 ewes), there was a basic difference in our study design, making further comparisons inappropriate; namely, we assessed the diagnostic performances of hoof temperatures at specific hoof regions rather than the performance of an average hoof temperature (as estimated by drawing a freehand border line to encompass the posterior part of the hooves) [40]. The same authors estimated the diagnostic performance of the average hoof temperature to detect footrot with a reported sensitivity and specificity of ca. 77.0% and 78.0%, respectively (threshold value: 9 °C above the average of the five coldest hooves in the flock) [40]. Similarly, Talukder, Gabai, and Celi [33] assessed the maximum temperature of the interdigital space for the diagnosis of footrot, with the optimal diagnostic performance being ca. 83.0% and 78.0% for the sensitivity and the specificity, respectively (threshold value: 36.4 °C).

Future studies on the thermal imaging of sheep hooves for the detection of hoof lesions could exploit the knowledge transfer and methodological aspects from studies in other farm animal species, but we cannot extrapolate conclusions from them. The most relevant applications of thermal imaging for the diagnosis of foot lesions have been recently studied in dairy cattle, and its reliability has been sufficiently documented [36,37,38,39,43]. The coronary band is the most-studied hoof site, and the measurement of its maximum temperature has been found to perform well for the diagnosis of foot lesions in dairy cattle [37,38,44,45]; in particular, Alsaaod and Büscher [37] reported a satisfying diagnostic performance of the temperature at the coronary band for the detection of digital dermatitis; laminitis; white line disease; sole ulcer; interdigital dermatitis; and hyperplasia pre- (sensitivity ca. 86.0%, specificity ca. 56.0%, and threshold value: 0.64 °C) or post-trimming (sensitivity ca. 80.0%, specificity ca. 83.0%, and threshold value: 1.09 °C). Likewise, Orman and Endres [45] found that the temperature at the coronary band could detect sole ulcers with a sensitivity ca. 78.0% and specificity ca. 65.0% (threshold value: 33.5 °C). Additionally, the difference between the maximum temperature of the coronary band and the skin above it has been found to be of diagnostic value for the detection of digital dermatitis lesions (ca. 89.0% sensitivity and 67.0% specificity; threshold value: 0.99 °C) [38].

Increased temperatures in the central and interdigital plantar regions have been observed in dairy cows with digital and interdigital dermatitis [46] and in cases of white line lesions, sole ulcers and hemorrhages, and horizontal and axial hoof cracks [39]. Similarly, lameness-related foot lesions were associated with an increased temperature of the plantar aspect of the foot between the heel bulbs and the accessory digits and the coronary band in dairy cows [44,47]. Moreover, in the same species, although the diagnostic performance of the maximum skin temperature of the plantar aspect of the pastern for the detection of hoof lesions has been evidenced (sensitivity 80.0% and specificity 73.0%; threshold value: 27 °C), differentiation between the foot lesions was not feasible [36].

Training of the thermal imaging camera operator to understand its limitations and the confounding factors is a crucial component for its efficient utilization for diagnostic purposes. This is achievable, as commercial thermal imaging cameras and their software are user-friendly, with low labor and operational demands. Moreover, the benchmarking capability is a key element when considering using thermal imaging to detect hoof lesions; therefore, a single operator needs to handle the camera and interpret the captured images. In our case, the operator was a veterinarian; however, thermal imaging could also be a farmer-friendly technology if the appropriate training is provided. Although the cost of purchasing a thermographic camera is relatively high, the expense can be justified, particularly in large flocks, given that the operational cost is minimal and the benefits from the effective herd health management outweigh the initial expense. In any case, the exploitation of thermal imaging for the detection of sheep hoof lesions does not imply the replacement of clinical examination and exploratory foot-trimming; on the contrary, it offers a rapid, on-farm, noninvasive, low-cost technique for the early detection of candidate animals that need to be further examined for possible underlying hoof pathologies on an evidential basis.

## 5. Conclusions

IRT is a user-friendly, noninvasive, and remote-sensing technology with the potential to be used on a regular basis for the screening and rapid assessment of hoof health in farm animals. In intensively reared dairy sheep, it provides a reliable and efficient tool for the detection of footrot and white line disease lesions. However, limitations, including environmental, operating, and operator factors, should be considered and appropriately addressed for its most effective exploitation under field conditions. Further studies are needed to elucidate the various thermal imaging application capabilities regarding hoof health assessments. In any case, IRT should be considered a supplementary tool for the early detection of animals with underlying hoof lesions, facilitating their selective physical examination and exploratory foot trimming to reveal and treat hoof diseases.

## Figures and Tables

**Table 1:** The Hosmer and Lemeshow tests, Omnibus tests of coefficients, and Nagelkerke R^2^ results for the set of three prediction models corresponding to the three hoof sites for (i) footrot and (ii) white line disease lesions.

	Hosmer and Lemeshow Test			Omnibus Test of Model Coefficients			Nagelkerke R^2^
	*Χ* ^2^	df	*p*	*Χ* ^2^	df	*p*	
Footrot							
DT-1	7.24	8	0.511	11.20	1	0.001	0.043
DT-2	9.20	8	0.326	8.26	1	0.004	0.032
DT-3	6.82	8	0.556	9.10	1	0.003	0.035
White line disease							
DT-1	5.23	8	0.732	12.20	2	0.002	0.013
DT-3	10.48	8	0.233	11.21	2	0.004	0.012

DT: Differences between the AT and the temperature at the three hoof sites: (1) the coronary band (DT-1), (2) the hoof heel (DT-2), and (3) the hoof sole (DT-3); df: degrees of freedom.

**Table 2:** Constants and regression coefficients of the predictor variables retained in the backwards stepwise regression models for the prediction of the occurrence of footrot and white line disease lesions.

		B	S.E.	*p*	OR	95% CI for OR	
						Lower	Upper
Footrot	Constant-1 ^a^	−6.52	0.694	0.000	0.00	na	na
	DT-1	0.20	0.061	0.001	1.23	1.09	1.38
	Constant-2 ^a^	−6.01	0.590	0.000	0.00	na	na
	DT-2	0.19	0.064	0.003	1.21	1.07	1.37
	Constant-3 ^a^	−6.01	0.591	0.000	0.00	na	na
	DT-3	0.22	0.071	0.002	1.25	1.09	1.44
White line disease	Constant-1 ^a^	−1.58	0.807	0.051	0.21	na	na
	DT-1	0.07	0.024	0.002	1.08	1.03	1.13
	BCS	−0.57	0.286	0.046	0.57	0.32	0.99
	Constant-2	−2.55	0.080	0.000	0.08	na	na
	Constant-3 ^a^	−1.75	0.821	0.033	0.17	na	na
	DT-3	0.09	0.031	0.003	1.10	1.03	1.16
	BCS	−0.50	0.287	0.084	0.61	0.35	1.07

DT: Differences between the ambient temperature and the temperatures at the three hoof sites: (1) the coronary band (DT-1), the hoof heel (DT-2), and (3) the hoof sole (DT-3); B: regression coefficient, S.E.: standard error, OR: odds ratio, CI: confidence interval, and BCS: body condition score; ^a^ validated models.

**Table 3:** The optimal efficiency thresholds for the predicted probabilities and the temperature differences between the studied hoof sites and the ambient temperature, sensitivity, and specificity, as estimated by the binary regression models for the total database, the training sample, and the validation sample.

	Footrot			White Line Disease	
	DT-1	DT-2	DT-3	DT-1	DT-3
Threshold value of the predicted probability	−4.23	−4.76	−4.32	−2.53	−2.66
Threshold values of DTs (°C)	11.3	6.5	7.9	10.0 *	6.4 *
Sensitivity (%)	51.9	83.3	51.9	64.8	73.1
Specificity (%)	79.7	47.8	76.4	51.0	40.7
Threshold value of the predicted probability (t.s.)	−4.23	−4.68	−4.44	−2.54	−2.66
Threshold value of DTs (°C) (t.s.)	11.3	6.9	7.4	10.4 *	6.4 *
Sensitivity (%) (t.s.)	56.0	76.0	64.0	67.5	75.9
Specificity (%) (t.s.)	80.1	52.3	69.1	49.4	39.4
Threshold value of the predicted probability (v.s.)	−4.22	−4.78	−4.24	−2.58	−2.60
Threshold value of DTs (°C) (v.s.)	11.3	6.4	8.3	9.9 *	7.1 *
Sensitivity (%) (v.s.)	51.7	89.7	51.7	72.1	62.3
Specificity (%) (v.s.)	81.0	45.5	80.2	44.7	52.2

DTs: Differences between the ambient temperature (AT) and the temperatures at the three hoof sites: (1) the coronary band (DT-1), (2) the hoof heel (DT-2), and (3) the hoof sole (DT-3); t.s.: training sample and v.s.: validation sample. * The body condition score is assumed constant and equal to 3.
